# Are Sulfates of the Hydroxyl Amino Acids Threonine and Serine Truly Extremely Rare or Do They Simply Escape Detection?

**DOI:** 10.3390/life16081325

**Published:** 2026-08-13

**Authors:** Simone König, Heather G. Marco, Gerd Gäde

**Affiliations:** 1 Service Unit Proteomics, Medical Faculty, University of Münster, 48149 Münster, Germany; 2 Department of Biological Sciences, University of Cape Town, Rondebosch, Cape Town 7701, South Africa; heather.marco@uct.ac.za (H.G.M.); gerd.gade@uct.ac.za (G.G.)

**Keywords:** adipokinetic hormone, protein sulfonation, labile modification, sulfotyrosine, sulfothreonine, sulfotransferase, sulfatase

## Abstract

Sulfonation is important for bioactivity across species—substrates range from small molecules to proteins, in which almost exclusively tyrosine sulfonation (sTyr) has been investigated. Although sulfoalkyl-hydroxyl amino acid residues in peptides and proteins have rarely been found, we will argue that it is highly likely that sSer and sThr are much more abundant than is currently evident. In the context of the recent detection of sThr-containing adipokinetic hormones in insects, the literature was screened to answer questions about the relative stability (lability) of the sulfate moiety at Tyr, Ser and Thr that may explain the historic abundance of sTyr reports; to ascertain if there are special grounds for finding sSer and sThr peptides in insects, and whether specific enzymes (sulfotransferases and sulfatases) involved in the biochemistry of sulfonation are documented in insects. Finally, we assessed detection methods for sThr and sSer-containing peptides to achieve greater accuracy. Our literature screening could not supply a special reason for the preferential formation of sThr/sSer in insects. Although the analysis of sulfonated molecules is challenging because of the lability of the sulfate group, sThr/sSer do not seem to be at a greater disadvantage compared to sTyr; it was even experimentally found that they are more stable than commonly reported. For untargeted studies of sulfoproteomes, special enrichment strategies are still required to identify these sulfonated peptides. Mass spectrometry is very valuable for the detection of the modification, but methods need to be adjusted, because sulfur trioxide is easily lost during measurement.

## 1. Introduction

**Figure 1:**
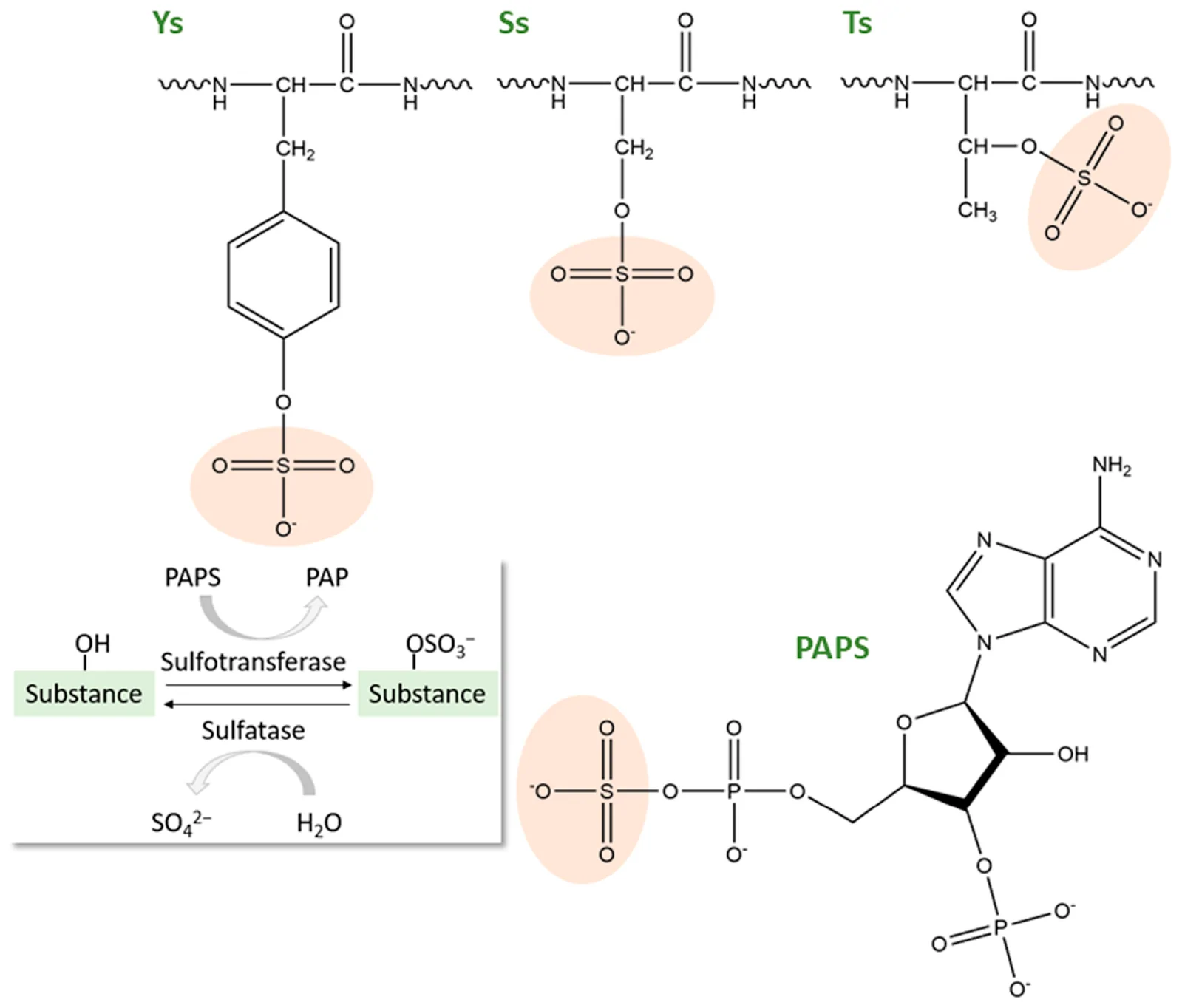
Schematic structures of the sulfonated hydroxyl amino acids and the sulfur donor PAPS (3′-phosphoadenosine-5′-phosphosulfate). Sulfotransferases (SULTs) catalyze sulfonation, while sulfatases (SULFs) remove the sulfate group. PAP: 3′-phosphoadenosine-5′-phosphate.

Sulfonation of endogenous molecules is a ubiquitous enzymatic process of great importance for the biological activity of substrates ranging from small compounds such as steroids to large glycoproteins (for reviews, see [1,2,3,4,5]). The term refers to the transfer of a sulfonate group (SO_3_^−1^) from the universal donor 3′-phosphoadenosine 5′-phosphosulfate (PAPS, Figure 1) to an acceptor molecule and can occur via ester and anhydride linkages (O-sulfonation), amide (N-sulfonation) and thioester (S-sulfonation) [1,6]. The transfer of SO_3_^−1^ to a hydroxyl or phenolic acceptor (O-sulfonation) represents the dominant cellular sulfonation reaction. The formation of the sulfono derivative is often, not quite accurately, referred to as sulfation, a non-enzymatic chemical process [6].

The large body of knowledge on the O-sulfonation of amino acids in proteins and peptides is essentially limited to the modification of tyrosine (sTyr) [4,5] and even that is considered one of the most understudied posttranslational modifications (PTMs) [7]. There is one singular publication from 2004 that deals with sulfoalkyl-hydroxyl amino acid residues in proteins [6]. Sulfonated serine (sSer) was found in a neuronal intermediate filament protein of the freshwater snail *Lymnea stagnalis* and in a cytoplasmic construct of the human orphan receptor tyrosine kinase Ror2, and sThr was detected in a cathepsin-C-like protein from the protozoan malaria parasite *Plasmodium falciparum.* These results from proteins of very different eukaryotic organisms and distinct cell compartments suggest that Thr and Ser sulfates may be widespread despite the lack of further data [6]. The authors extensively validated their findings, including the synthesis and comparative analysis of the modified peptides. This was not the case in other published work on this topic, where sSer was detected in the murine dioxin receptor using MALDI-TOF/TOF mass spectrometry (MS) [8].

We recently contributed our findings of sThr in three beetle adipokinetic hormones (AKHs) across several species [9]. AKH peptides are important metabolic regulators in insects. They are responsible for the release of diacylglycerols and/or trehalose from the fat body during extensive locomotory activity (for a review, see [10]). They are mostly eight to ten amino acid residues long with modified termini (N-terminal pyroglutamic acid and an amidated C-terminus). Typically, one or two AKHs are synthesized in and released from the cephalic neurohemal organs, the corpora cardiaca. We detected sThr in position 6 in the AKHs of 11 different species of two beetle subfamilies (Cetoniinae and Dynastinae) of the large superfamily of Scarabaeoidea (dung beetles, rhinoceros beetles, flower beetles) and in one bug species (family Coreidae) using high-resolution MS and validated them using synthetic analogs. Most PTMs in AKHs described so far occur at the sixth residue and include Thr phosphorylation [11], Pro hydroxylation [12,13] and a possible Pro *cis*–*trans* isomerization [14]; hypertrehalosaemic and hyperprolinaemic (AKH) bioactivity was demonstrated for some of them [13,15,16].

Even after an extensive research of the body of knowledge, it appears that the above-mentioned findings constitute the only information about sSer or sThr PTMs in proteins or peptides—in a great contrast to sTyr (see below, Section 2, Section 3 and Section 4). It should be noted that artifactual sulfation of Thr was reported during peptide synthesis [17,18,19] and, additionally, of Ser and Tyr during silver staining, resulting from the use of sodium thiosulfate [20].

Based on our prior experiments hunting this modification in beetle AKHs (as detailed in [9]), we had no difficulty finding the sulfonated AKH peptides in the recent experiments because their abundance matched that of unmodified AKHs for which we have extensive analytical experience [21]. In most cases, the sThr-containing peptide was dominant, and the unmodified form could hardly be detected [9]. Although we also noted some lability of the sulfate moiety during MS-based analysis, it did not impact so heavily on peptide detection as one might have assumed from the reports that stress the challenges associated with preserving the sulfate group during sample preparation and during analysis, including acid lability, thermal instability, and the easy loss of SO_3_ in MS sequencing experiments [7].

Therefore, we took a fresh look at the publicly available scientific resources, including sequence databases, to find answers to the following questions:

1. How labile is the well-known sulfate moiety at Tyr? Are sSer/sThr residues even more labile, and could that be the reason for their rarely reported findings?
2. Is there a special reason why Thr/Ser sulfonation has been found in quite a few insects, or is its finding simply a matter of research focus?
3. What is known about the modifying and degrading enzymes—sulfotransferases (SULTs) and sulfatases (SULFs)—in insects in this context?
4. Can we improve the detection methods of sThr/sSer-containing peptides?

## 2. How Labile Is sTyr and Are sSer and sThr Even More Labile and Thus Not Discoverable?

A 2024 review featuring the analysis of sTyr next to other uncommon PTMs [7] presents a valuable overview of the knowledge acquired so far. A major difficulty that is stressed is the neutral loss of sulfur trioxide from sTyr during the enrichment of sulfopeptides at low pH and in the ion source of the mass spectrometer during heated electrospray ionization or MALDI, as substantiated by several publications from different groups. Furthermore, several scientists, including ourselves [7,9] observed the phenomenon that SO_3_ is quickly and completely lost from the modified peptide during collision-induced dissociation (CID) so that no sulfate-containing fragment ions can be detected for the assignment of the modified amino acid residue—a feature that contrasts with phosphorylated peptides. We directly compared a sulfonated and a phosphorylated AKH of the same amino acid sequence using high-resolution MS [9] and found this differing property rather convenient as an additional parameter to distinguish sThr- and pThr-containing AKHs, which differ in mass by only 9.4 mmu.

In our experience [9], the sulfonated AKHs were not very labile when kept in 0.1% formic acid containing 5% acetonitrile (pH 2.3), which we typically use for liquid chromatography (LC) separation coupled to MS. Even when the tissue extracts were remeasured following a freeze–thaw-cycle, there did not seem to be any detectable loss of the sThr-containing AKH toward the unmodified form. Thus, as a test, we kept the synthetic sulfated Pacsi-AKH (for peptide structures, see [9]) in the autosampler at 4 °C and measured a time course of eight days by reinjecting from the same sample into the LC-MS system. For this period, we observed no decrease in peak intensities. In fact, the influence of work on unrelated samples in between on the nanoLC-MS system was a greater factor than the effects of substance degeneration (Supplementary Figure S1). Similar results were obtained for sulfated Penid-AKH measured in 1% aqueous acetic acid/90% methanol (pH 3.1), which is often used for AKH extraction, over the period of five days and, in a repeat experiment with a fresh sample, nine days (Supplementary Figure S1). This result agrees with data from stability experiments of other authors with sTyr-containing peptides at low pH, where the authors found only marginal hydrolysis of gastrin-17 in 0.5% trifluoroacetic acid solution (pH 1.2) at room temperature [22]. The hydrolysis rate seemed to depend on the primary amino acid composition of their test peptides, but, generally, it was concluded that sTyr is more stable under normal protein purification conditions than is usually assumed [22]. There are no comparable hydrolysis data for sThr/sSer-containing peptides. Although sulfate attached via aliphatic oxygen is thought to be more reactive compared with phenolic oxygen, the results for monoalkyl sulfates attest to them a marked resistance to hydrolysis [23]. Nevertheless, for screening experiments, which require the enrichment of the sulfoproteome or of sulfopeptides, care is required. It was repeatedly reported that the low pH 2 used in phosphopeptide enrichment is not tolerated by sulfopeptides, thus requiring alternative isolation strategies [7].

We need to acknowledge that there was a massive loss of the sulfated peptide during the synthesis process of the synthetic peptides we made for our earlier validation work; a much lower yield was observed for the sulfated peptide than for the parent peptide, likely due to acidic steps in the procedure [9].

In summary, sulfonated hydroxyl amino acids are more stable than is commonly assumed for routine laboratory procedures, but their stability may vary considerably under specific experimental conditions and should always be tested. Preliminary experiments with synthetic sulfated analogs of the peptides of interest are highly recommended to instill confidence in the chosen preparation workflow, analytical setup and instrumental parameters (see our experiments with synthetic sulfated AKHs [9] and reference [6]).

## 3. Are Insects Special Regarding Thr/Ser Sulfonation and What Is Known About Their Modifying and Degrading Enzymes, SULTs and SULFs?

Sulfur and the synthesis of activated sulfate used in sulfonation pathways are essential for all organisms. These pathways, modifying a great variety of different substrates, involve the activity of SULTs and SULFs (Table 1). Mutations in genes encoding these enzymes can cause developmental aberrations in humans and dwarfism in plants; the loss of function of the enzymes can be fatal in flies and worms [24]. There are several excellent reviews available on the current state of knowledge in this regard, with a strong focus on the respective processes in humans [1,24,25,26], including the latest advances in the area of SULTs [27,28] (for plants [29], fungi [30]) and SULFs [31].

SULTs in insects have not been heavily researched. An early esterase screening (1970) in the thoracic muscle of the American cockroach, *Periplaneta americana*, for use as a possible taxonomic indicator, included SULFs [32]. In 1998, it was demonstrated that retinol dehydratase of the fall armyworm moth *Spodoptera frugiperda* could catalyze the transfer of the sulfonate moiety to small phenolic compounds [33], making it a unique enzyme among members of the SULT superfamily (see nomenclature for cytosolic SULTs [34]). It apparently functions as a retinol dehydratase, with sulfonation being an intermediary catalytic reaction. This enzyme has been cloned and characterized [35]. Additionally, from the gut tissue of the southern armyworm *Spodoptera eridania*, an enzyme system that catalyzed the sulfonation of p-nitrophenol and nine steroids such as cholesterol and α-ecdysone was obtained [36] followed by proof for aryl SULF activity in the late larval development [37,38]. In the honey bee, *Apis mellifera,* aryl SULF activity in semen was associated with the penetration and/or fertilization of the insect egg [39]. In mosquitoes, a SULT was shown to be crucial for development and reproductive success and represents a key target for disrupting survival [40]. Low molecular weight molecules and xenobiotics are acceptors for sulfonation by a cytosolic SULT of the malaria vector *Anopheles gambiae* [41]. SULTs of the silkworm *Bombyx mori* modify small compounds such as xanthurenic acid and pentachlorophenol [42] and are involved in polyphenol metabolism [43]. The authors suggest a role for SULTs in detoxification and in the molecular mechanism responsible for host plant selection in lepidopteran insects. Moreover, SULFs in the blood of silkmoths *Hyalophora cecropia* and *Samia cynthia* have been associated with insect development [44].

By now, the sulfuryl transfer reaction catalyzed by cytosolic SULTs is accepted as one of the major conjugating pathways responsible for the detoxification and subsequent elimination of xenobiotics; however, the functional characterization of insect SULTs is still limited. Knowledge about these processes in insects is of profound practical importance in pest control. The knockdown of a cytosolic SULT in the red flour beetle, *Tribolium castaneum*, significantly increased the susceptibility to the insecticide deltamethrin [45].

Investigations of the whole genome of the diamondback moth *Plutella xylostella*, a basal lepidopteran species that is the worst pest species of cabbage plants (Brassicaceae) in the world, characterized these detoxification-related genes and genes involved in the development of insecticide resistance [46]. The focus in that regard was on glucosinolate SULF activity, which is considered a central host adaptation strategy in *P. xylostella* against the well-researched glucosinolates–myrosinase defense system in cruciferous plants (for reviews, see [47,48]). The nonsteroidal inhibitor of steroid SULF, isosustat (aryl sulfamate ester class of drugs), inhibits glucosinolate SULF in this species and thus impairs the ability of *P. xylostella* to detoxify the glucosinolate–myrosinase system, leading to the systematic accumulation of toxic isothiocyanates in larvae, thereby severely affecting feeding, growth, survival, and reproduction [49]; accordingly, the substance was suggested for pest control. SULF activity was also studied for host glucosinolate adaptation of the lepidopteran pest, the cotton bollworm *Helicoverpa armigera* [50,51], the invasive whitefly, *Bemisia tabaci* [52,53,54], the swede midge, *Contarinia nasturtii* [55], the sawfly, *Athalia rosae* [56,57], and the desert locust, *Schistocerca gregaria* [58].

Protein sequence information is available in the large public Uniprot archive for additional insect SULTs and SULFs, mostly derived from sequencing projects. Both types of enzymes were found in 16 species; three further species had only a SULT and four had only a SULF (Table 1). Protein alignment analysis demonstrated a similarity of more than 50% with retinol dehydratase from *S. frugiperda* of many of the insect SULTs from different species. When performing this analysis with the SULF from *P. xylostella*, only Uniprot entries for *Plutella* species showed the same level of sequence similarity.

In summary, both SULTs and SULFs have been described in insects, but their substrates so far seem to be mostly small molecules. While the presence of sTyr in proteins and peptides has been reported (yolk protein 2 of *D. melanogaster* [59], sulfakinins, e.g., in the cockroaches *Leucophaea maderae* [60] and *P. americana* [61,62]; for more on sulfakinins, see [63]), a tyrosyl-SULT (TPST) has only been discussed for *D. melanogaster*. The fly has only one TPST gene, while most species have two [64,65]. Present evidence assigns TPST as an integral membrane protein to the trans-Golgi network [4]. While cytosolic SULTs have very broad substrate specificities [4], the crystal structure of human TSPT-2 suggests the need for acidic residues in the catalytic site [66]. In another mechanistic model of this human enzyme, independent binding of substrates to two distinct sites is allowed and involves the formation of a sulfonated enzyme-covalent intermediate [67]. The sulfoAKHs known to date do not have the acidic amino acid residues that are supposed to be needed for enzymatic sulfonation. Possibly, different mechanisms may apply to their modification.

**Table 1:** Available information on insect SULTs and SULFs from Uniprot (searched 3 February 2026). Entries in gray refer to our finding of Thr-sulfonated peptides [9], for which no enzymes have been investigated so far.

Order	Suborder	Infraorder	Family	Species	Common Name	Uniprot		Refs. Here		
						SULT	SULF	SULT	SULF	sThr
Blattodea			Blattidae	*Periplaneta americana*	American cockroach				[32]	
Coleoptera	Polyphaga	Cucujiformia	Cerambycidae	*Aromia moschata*	Musk beetle	x	x			
			Cerambycidae	*Exocentrus adspersus*	Longhorn beetle	x	x			
			Cerambycidae	*Molorchus minor*	Beetle	x	x			
			Cerambycidae	*Rhamnusium bicolor*	Beetle	x	x			
			Chrysomelidae	*Diabrotica virgifera*	Western corn rootworm	x	x			
			Curculionidae	*Dendroctonus obsoletus*	Bark beetle	x	x			
			Tenebrionidae	*Tribolium castaneum*	Red flour beetle			[45]		
		Scarabaeiformia	Scarabaeidae	11 species	Scarab beetles					[9]
Diptera	Brachycera		Drosophilidae	*Drosophila melanogaster*	Fruit fly	x				
			Tabanidae	*Tabanus bromius*	Band-eyed brown horsefly	x				
	Nematocera		Cecidomyiidae	*Contarinia nasturtii*	Swede midge				[55]	
			Ceratopogonidae	*Culicoides obsoletus*	Midge	x				
			Corethrellidae	*Corethrella appendiculata*	Midge	x	x			
			Culicidae	*Anopheles gambiae*	Mosquito			[41]		
Ephemeroptera			Baetidae	*Cloeon dipterum*	Mayfly		x			
Hemiptera	Auchenorrhyncha		Cicadellidae	*Cuerna arida*	Sharpshooter	x	x			
			Cicadellidae	*Graphocephala atropunctata*	Blue-green sharpshooter	x	x			
			Cicadellidae	*Homalodisca liturata*	Smoketree sharpshooter	x	x			
			Clastopteridae	*Clastoptera arizonana*	Arizona spittlebug	x	x			
	Heteroptera		Coreidae	*Holopterna alata*	True bug					[9]
			Nepidae	*Ranatra chinensis*	Chinese water scorpion	x	x			
	Sternorrhyncha		Aleyrodidae	*Bemisia tabaci*	White fly				[52,53,54]	
			Coccidae	*Parthenolecanium corni*	European fruit scale	x	x			
Hymenoptera			Apidae	*Apis mellifera*	Honey bee				[39]	
			Athalia	*Athalia rosae*	Saw fly				[56]	
Lepidoptera			Bombycidae	*Bombyx mori*	Silk moth	x	x	[42]		
			Noctuidae	*Helicoverpa armigera*	Cotton bollworm				[50,51]	
			Noctuidae	*Spodoptera eridania*	Southern armyworm			[36]	[37]	
			Noctuidae	*Spodoptera frugiperda*	Fall armyworm		x	[35]		
			Plutellidae	*Plutella australiana*	Moth		x			
			Plutellidae	*Plutella xylostella*	Diamond back moth				[46,47,48,49]	
			Saturniidae	*Hyalophora cecropia*	Silk moth				[44]	
			Saturniidae	*Samia cynthia*	Silk moth				[44]	
			Sphingidae	*Manduca sexta*	Tobacco hawk moth	x	x			
			Yponomeutidae	*Yponomeuta cagnagella*	Spindle ermine moth		x			
Orthoptera	Caelifera		Acrididae	*Schistocerca gregaria*	Desert locust				[58]	
Thysanoptera			Thripidae	*Thrips palmi*	Melon thrips	x	x			

For the sulfoAKHs, we had obtained proof of their bioactivity in early experiments, which triggered the subsequent study on the nature of the modification (for details, see [9]); in general, it is difficult to measure the biological activity of hormones conspecifically in insect species that are not available from breeders in large numbers—where collecting permits and the seasonal abundance of insects in the wild may serve as barriers. However, we note the high concentration of the sThr-AKHs in the corpora cardiaca relative to their unmodified (parent) form [9], which suggests a biological relevance. Active cholecystokinin (CCK), for instance, is a fully sulfonated peptide and gastrin is approximately 50:50 sulfonated:not sulfonated [1]. Bioactivity has been shown for a pThr^6^-AKH [11] so that it can be speculated that the sThr^6^-AKHs [9] may also be active. SULF activity has already been associated with hormonal processes in reproduction, development, and survival in insects [39,40,44].

**Figure 2:**
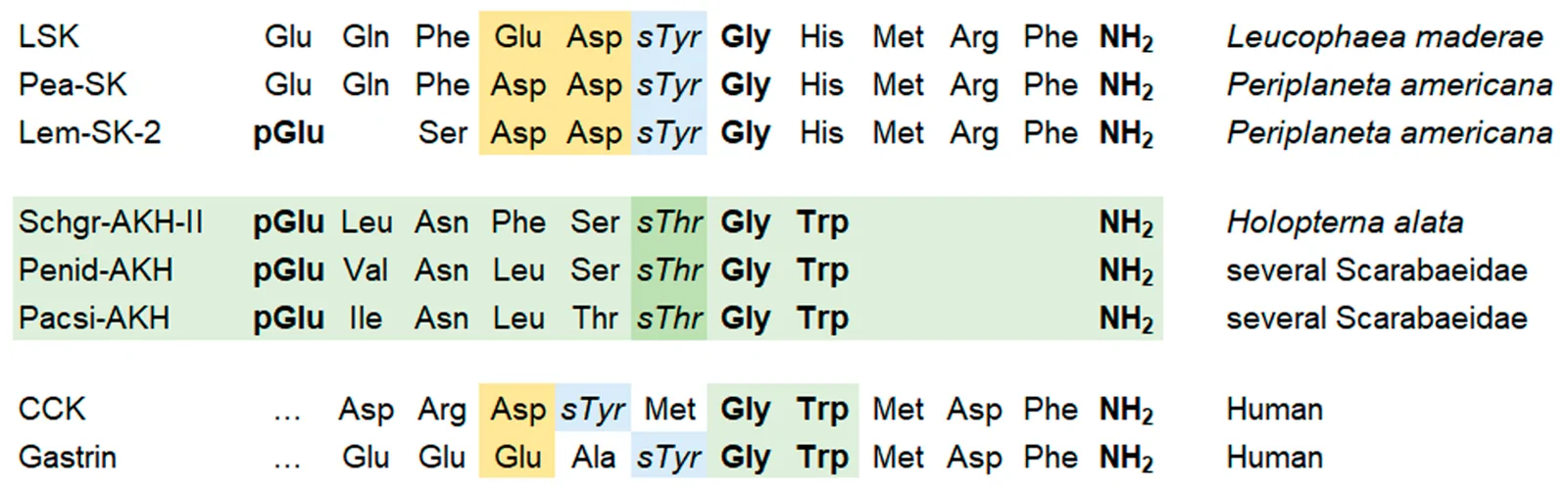
Comparison of terminal sequences of sTyr human neuroendocrine peptides CCK and gastrin [1], cockroach sulfakinins [60,61,62] and the known sThr-AKHs of insects (highlighted in light green) [9]. They share a G- or GW-motif close to the modification site as well as amidation which is crucial for bioactivity. Shared features are printed in bold, modified sites in italic (sTyr—light blue, sThr—dark green). Acidic amino acids possibly needed for sulfonation [66] are colored orange. Modified after [9].

The comparison of known neuroendocrine sTyr-sulfopeptides CCK and gastrin, as well as cockroach sulfakinins, reveals interesting similarities to the sThr-AKHs, including a G- or GW motif next to the sulfosite, C-terminal amidation, and in some cases N-terminal pyroglutamic acid (Figure 2) [63,68]. Sulfakinin receptors are similar to CCK receptors in mammals and they belong, like AKH receptors, to the G-protein-coupled receptors [1,59,60,68]. For AKH receptors, Uniprot counts 112 sequence entries for 25 species, and some have been cloned and tested in functional in vitro assays (e.g., *Coccinella septempunctata* [69], *Tribolium castaneum*, *Schistocerca gregaria* [70], *Carausius morosus* [71]).

Conclusively, our finding of sThr-AKHs seems to be the fortuitous result of the deep-digging and persistent research activity in this area over the past 50 years [9,10]. It is highly likely that additional peptides and proteins with sulfonated hydroxyl amino acids other than Tyr will be detected. No obvious reason for the opposite could be found, and hydroxyl-containing amino acids are primary sites for dynamic PTMs in proteins due to a combination of chemical reactivity, structural accessibility, and functional impact. The oxygen atom in the hydroxyl group possesses two lone pairs of electrons, making it an effective nucleophile. Enzymes can easily catalyze reactions where this oxygen attacks electrophilic centers. Covalent bonds formed via the oxygen atom are chemically sufficiently stable under physiological conditions and are easily cleaved by dedicated hydrolytic enzymes. This property makes them ideal for temporary, regulatory switches. Because Ser, Thr, and Tyr are polar (or amphipathic, in the case of Tyr), they naturally cluster on the exposed surfaces of folded proteins where enzymes can access them [72,73,74].

## 4. Can the Detection of sThr/sSer Containing Proteins and Peptides Be Improved?

Both sulfate and phosphate groups introduce negative charges into biological macromolecules. They are superficially similar but differ in the valency of the central atom (P or S) with consequences regarding the charge and solubility of the respective compounds; sulfate introduces charge without exerting pronounced intramolecular or intermolecular effects compared with phosphate (for a detailed comparison of phosphorylation and sulfonation, see [26]). Analytical tools successfully employed for phosphorylation analysis may thus not be useful for the detection of sulfonation, including enrichment strategies for phosphopeptides [7]. Initially, protein sulfonation was investigated with [^35^S]-labeling and other tools probing sTyr [7]. Since then, MS-based strategies have gained influence and have been used for the analysis of hundreds of protein modifications, including phosphorylation and sulfonation. Both modifications require, however, special attention because of their low abundance and their lability in analytical workflows [7,9,75]. Moreover, sulfonated and phosphorylated peptides differ only slightly in mass, thereby requiring high-resolution MS for their distinction. In shotgun proteomics experiments, based on the software settings, both modifications may be confused, leading to misassignments by automated spectrum analysis. A recent bioinformatic screen of phosphoproteomic datasets for the misassignment of sTyr as pTyr concluded that the misidentification of sulfonation can occur but appears relatively rare [76].

Depending on the analytical question and sample matrix, MS is, despite the lability of the sulfo-moiety, an extremely useful tool for identification of the modification and its validation even in positive ion mode, as we demonstrated with the de novo sequencing of peptide hormones [9]. Still, it cannot be used for the assignment of the modified amino acid residue as it would be possible to some extent for phosphopeptides. To that end, Edman degradation sequencing or the new methods of nanopore sensing provide possible alternatives [77]. With the latter, sulfonation can be clearly detected and distinguished (>90%) from phosphorylation on the same residue [78].

The situation is different in global approaches to the sulfoproteome. There is no way around specific enrichment in that context, because otherwise the sulfopeptides or sulfoproteins will “drown” in the bulk of undesired matrix. The above-mentioned 2024 review [7] summarized the efforts to that effect, including weak anion exchange in combination with enzymatic and chemical modification of the proteins, antibody-based affinity enrichment, and immobilized metal affinity chromatography (IMAC), none of which provided a perfect solution, but offered some results, nonetheless. The same can be said for the use of different MS fragmentation methods and the choice of negative polarity instead of positive electrospray ionization [7]. This was again highlighted in a 2023 MS-based workflow for sTyr-peptide characterization, which incorporated optimized Zr^4+^-IMAC and TiO_2_ enrichment strategies and used an array of fragmentation regimes (CID, HCD, ETD, UVPD) [79].

In summary, tried and tested methods for sulfoprotein and sulfopeptide analysis are available to get started (see examples above). However, the approach that can be taken will depend on the requirements of the individual project, as well as the capabilities of the available instrumentation. There is no one-size-fits-all solution, and even the use of unfavored techniques over suggested methods, such as the use of positive instead of negative ESI may prove successful. It cannot be stressed enough that preliminary experiments with synthetic sulfated peptides, which are similar or identical to the compounds under investigation, are the best way to evaluate sulfate losses during the workflow [9]. Collaboration with an organic chemist seems to be still required for this purpose, because commercial offers are limited.

## 5. Conclusions

The sulfoproteome, despite its importance, is heavily underresearched, and the available studies have focused on sTyr. The still rare but increasing findings of sulfonated alkyl hydroxyl amino acid residues in proteins and peptide hormones suggest the presence of more yet undiscovered, biologically and functionally important, modified forms. While their analysis can be a challenge because of the lability of the sulfate group, it is not a hopeless case. In fact, several studies have shown that sulfated compounds are more stable in routine lab procedures than was expected. We provide an overview of the state of knowledge regarding the analyses, challenges and findings of sulfo hydroxyl amino acid residues in proteins and peptides.

## Data Availability

Data are available upon request from the corresponding author.

## Supplementary Materials

The following supporting information can be downloaded at: https://www.mdpi.com/article/10.3390/life16081325/s1, Figure S1: Stability nanoLC-MS experiments (Synapt G2 Si coupled to MClass UPLC, Waters Corp.) with synthetic AKHs. Injection of 0.1 µL of 1 pmol/µL solution per time point. Given is the MS intensity in counts. No continuous decrease in peak intensities was observed; the influence of work on un-related samples on the nanoLC-MS system in between stability measurements was greater than effects of substance degeneration. Intensity values vary within technical error.

## Abbreviations

The following abbreviations are used in this manuscript:

AKH	Adipokinetic hormone
CCK	Cholecystokinin
CID	Collision-induced dissociation
ETD	Electron transfer dissociation
HCD	Higher-energy C-trap dissociation
UVPD	UV photodissociation
MS	Mass spectrometry
SULT	Sulfotransferase
SULF	Sulfatase
IMAC	Immobilized metal affinity chromatography
LC	Liquid chromatography
PAP	3′-phosphoadenosine-5′-phosphate
PAPS	3′-phosphoadenosine-5′-phosphosulfate)
MALDI-TOF	Matrix-assisted laser desorption ionization time-of-flight
