# Supplementary material for: Are Sulfates of the Hydroxyl Amino Acids Threonine and Serine Truly Extremely Rare or Do They Simply Escape Detection?
Source: Life (Basel). 2026 Aug 13;16(8):1325. doi: 10.3390/life16081325 (PMC13514629; doi:10.3390/life16081325)
Supplement: Supplementary file 1 [file life-16-01325-s001.zip › life-4415450-supplementary.pdf]

life

MDPI

Perspective

Are sulfates of the hydroxyl amino acids threonine and serine truly extremely rare or do they simply escape detection?

Simone König *[1], Heather G. Marco [2] and Gerd Gäde [2]

[1] Service Unit Proteomics, Medical Faculty, University of Münster, 48149 Münster, Germany; koenigs@uni-muenster.de
[2] Department of Biological Sciences, University of Cape Town, ZA-7701 Rondebosch, Cape Town, South Africa; gerd.gaede@uct.ac.za; heather.marco@uct.ac.za
* Correspondence: koenigs@uni-muenster.de

Pacsi-AKH, 0.1% FA, 5% ACN		
Time	Intensity e5	
0	3,2	
1h	3,6	
3d	0,8	Pressure problem, repair
3d 1h	4,6	
8d	4,1	

Penid-AKH, 90% MeOH, 1% Hac					
Time	Intensity e4		Repeat	Day	Intensity e4
0	2,6			0	2,9
2d	3,8			2	2,3
2d 2h	3,8			3	4,4
3d	4,8			4	1,7
4d	1,3			7	5,1
5d	5,4			8	7,4
				9	7,8

Figure S1. Stability nanoLC-MS experiments (Synapt G2 Si coupled to MClass UPLC, Waters Corp.) with synthetic AKHs. Injection of 0.1 µl of 1 pmol/µl solution per time point. Given is the MS intensity in counts. No continuous decrease in peak intensities was observed; the influence of work on unrelated samples on the nanoLC-MS system in between stability measurements was greater than effects of substance degeneration. Intensity values vary within technical error.

Disclaimer/Publisher's Note: The statements, opinions and data contained in all publications are solely those of the individual author(s) and contributor(s) and not of MDPI and/or the editor(s). MDPI and/or the editor(s) disclaim responsibility for any injury to people or property resulting from any ideas, methods, instructions or products referred to in the content.
